# Evaluation of characteristics of CD44^+^CD117^+^ ovarian cancer stem cells in three dimensional basement membrane extract scaffold versus two dimensional monocultures

**DOI:** 10.1186/1471-2121-14-7

**Published:** 2013-01-31

**Authors:** Junsong Chen, Jing Wang, Dengyu Chen, Jie Yang, Cuiping Yang, Yunxia Zhang, Hongyi Zhang, Jun Dou

**Affiliations:** 1 Department of Pathogenic Biology & Immunology, Medical School, Southeast University, Dingjiaqiao 87, Nanjing, 210009, China; 2 Department of Gynecology & Obstetrics, Zhongda Hospital, Medical School, Southeast University, Dingjiaqiao 87, Nanjing, 210009, China; 3 Jiangsu Simcere Pharmaceutical R&D center, No.699-18 XUANWU Ave, Nanjing, 210042, China

**Keywords:** Three-dimensional culture, Epithelial ovarian cancer, Cancer stem cells, Chemoresistance

## Abstract

**Background:**

Cancer stem cells (CSCs) are thought to be capable of surviving conventional chemotherapeutic treatments because the cells have more resistant to anticancer drugs than common cancer cells. Most *in vitro* studies in experimental cancer cells have been done in a two-dimensional (2D) monocultures, while accumulating evidence suggests that cancer cells behave differently when they are grown within a three-dimensional (3D) culture system.

**Results:**

The CD44^+^CD117^+^cells isolated from human epithelial ovarian cancer SKOV-3 cell line using magnetic-activated cell sorting were found to grow faster than the SKOV-3 cells in the 3D culture and in the nude mice. Anticancer drugs 5FU, docetaxel, cisplatin, and carboplatin were seen to inhibit growth of the CD44^+^CD117^+^ cells by 50% in the 2D culture with IC_50_ concentration, whereas, in the 3D culture, the four drugs inhibited the cell growth by only 34.4%, 40.8%, 34.8% and 21.9% at 3D one, respectively. Effect of paclitaxel on the CD44^+^CD117^+^cell viability indicated that fewer cells underwent apoptosis in 3D culture than that in 2D one. In addition, anticancer drugs markedly increased the expression of ABCG2 and ABCB1 of CD44^+^CD117^+^cells in 3D culture.

**Conclusion:**

Our assay demonstrated that human epithelial ovarian cancer CD44^+^CD117^+^cells possessed the properties of CSCs that exhibited more chemoresistance in the 3D culture than that of in 2D one. The 3D culture provides a realistic model for study of the CSC response to anticancer drugs.

## Background

Ovarian cancer is the number one leading cause of death among gynecologic malignancies. This is due mainly to the prevalence of this undetected metastatic disease as over 90% of malignant tumors are epithelial ovarian cancer (EOC). Although the standard therapy of optimal cytoreductive surgery followed by the systemic chemotherapy with platinum-paclitaxel (PTX) has resulted in a complete remission in over 70% patients with EOC, the overall 5-year survival rate has been less than 30%. Most patients will relapse within 2 years after the treatment as development of resistance to platinum-PTX-based chemotherapy has developed. Moreover, recurred tumors tend to became unresponsive to additional chemotherapy [1-3].

Cancer stem cells (CSCs) represent a distinct subpopulation of the tumor cells that play an important role in the tumor initiation, progression, metastasis, chemoresistance and relapse [4,5]. Recent advance in *in vitro* experiment has shown more resistance to treatment with cisplatin (CDDP) and PTX by EOC CSCs than by their differentiated progeny [6]. It is believed that the cytotoxic effects of chemotherapy kill most cells in a tumor but CSCs are leave behind; this might be an important mechanism of the observed resistance to the treatment. CSCs are even more chemoresistant and more aggressive than their original tumor cells [7-9].

In one study [10], EOC CSCs from primary human ovarian tumors were isolated and characterized; the cells with a high expression of CD44 and CD117 molecules became highly tumorigenic and capable of re-establishing their original tumor hierarchy when 100 CD44^+^CD117^+^cells were injected into the nude mice that has been propagated with the original tumors. The CD44^+^CD117^+^ cells possess the properties of CSCs. Targeting CSCs could be a highly valuable therapy for the recurrent and chemoresistant EOC disease. However, the vast majority of studies that has identified cancer-associated genes and therapeutic targets has used adherent cells grown in a traditional two-dimensional (2D) cell culture system. The 2D system has limited capability of accurately recreating the *in vivo* tumor environment that plays a key role in tumor cell growth [11,12].

The culture of tumor cell lines in a three-dimensional (3D) scaffolds has been increasingly employed as it mimics the *in vivo* tumor environment better than the standard method of 2D method on a plastic substrate. The 3D cell culture *in vitro* bridges the gap between 2D cell culture *in vitro* and tumors *in vivo*. However, the 3D environment must be mimicked during the course of cell-based studies to provide the most accurate translation to animal models and clinical investigations [12,13]. In addition, the interactions between tumor cells and stroma in the use of 3D environment are considered critical for the growth, the drug resistance and the invasiveness of epithelial tumors because the composition of the extracellular environment in the 3D culture enables researchers to more accurately predict the *in vivo* response to chemotherapeutic therapy for the ovarian cancer [14,15]. A recent study reported that the *in vivo*-like tumor stromal 3D system was used to investigate the characterization of the specific traits of CSCs in the breast cancer MCF-7 cell line, including the self-renewal that was assessed by their clonogenic growth, its expression of specific surface markers, and drug resistance [16].

In this study, we employed a porous 3D basement membrane extract (BME) scaffold that mimicked the *in vivo* environment to evaluate the effect of anticancer drugs 5FU, Docetaxel (DXT), CDDP and Carboplatin (CBP), respectively on EOC CD44^+^CD117^+^ CSCs that were isolated from human SKOV-3 cell line in a 3D *in vitro* environment versus a 2D *in vitro* environment. In comparison with the drug responsiveness of CD44^+^CD117^+^CSCs in a plastic substrate 2D environment, the cells cultured within 3D BME scaffold showed more responses to anticancer drugs. Our findings may make significant contribution to growing EOC stem-like cells in the 3D culture model for anticancer drug screening, which may help develop valuable therapeutic approaches to treat ovarian cancer.

## Methods

### Cell line and animal

The human EOC SKOV-3 cell line for this study was from ovarian cancer patient of origin, a well-established ovarian cancer model system; the cell line was purchased from the Cellular Institute(in Shanghai, People’s Republic of China), and was maintained in the Dulbecco’s Modified Eagle Medium (DMEM, Invitrogen, NY, USA) supplemented with 10% fetal bovine serum plus 1% penicillin and streptomycin admixture. Athymic nude mice (BALB/c-nu, female) of 4-5 weeks of age were acquired from the Animal Center of Shanghai of China. The mice were raised under sterile conditions in the animal facilities of the Experimental Animal Center, Jiangsu Simcere Pharmaceutical R&D center, Nanjing, China. All the experiments on animals were conducted following the guidelines of the Animal Research Ethics Board of Southeast University. Full details of the study approval can be found under the approval ID, 20080925

### Isolation of EOC CSCs and identification of CSC phenotype

The CD44^+^CD117^+^cells were sorted from the SKOV-3 cell line by using the magnetic-activated cell sorting (MACS, Miltenyi Biotec., Bergisch Gladbach, Germany). First, CD44^+^subsets were isolated by using mouse antihuman CD44 antibody coupled to magnetic microbeads (Miltenyi Biotec., Bergisch Gladbach, Germany) and followed by the magnetic column selection or depletion. Second, resulting cells were then depleted of CD117^−^subsets by using mouse antihuman CD117 antibody coupled to magnetic microbeads (Miltenyi Biotec., Bergisch Gladbach, Germany), and we named CD44^+^CD117^+^cells for EOC cancer stem-like cells(EOC CD44^+^CD117^+^CSCs) [10]. The isolated cells were placed in stem cell culture medium by resuspension in serum-free DMEM/F12 supplemented with 5 μg/mL insulin (Sigma-Aldrich, Missouri, USA), 20 ng/mL human recombinant epidermal growth factor (Invitrogen, CA, USA), 10 ng/mL basic fibroblast growth factor (Invitrogen, CA, USA) and 0.5% bovine serum albumin (Sigma-Aldrich, Missouri, USA) [17,18]. The enrichment and recovery of CD44^+^CD117^+^CSCs were further identified by using fluorescence microscopy (Olympus X71, Japan) and a FC500 flow cytometer (FCM, Beckman Coulter, USA). Briefly, A total of 2 × 10^5^ CD44^+^CD117^+^CSCs and SKOV-3 cells were suspended in PBS and labeled with anti-Human/Mouse CD44 FITC 1:100 (eBioscience, CA, USA), and anti-Human CD117 PE 1:20 (eBioscience, CA, USA) antibodies for immunofluorescence detection. Equal number of the cells cultured in stem cell culture medium was analyzed by FCM with Beckman Coulter CellQuest software on the 2nd day after sorting [19].

### *In vivo* xenograft experiment

CD44^+^CD117^+^CSCs, SKOV-3 cells and CD44^−^CD117^−^cells were respectively resuspended in 40 μL 1:1 PBS/Rat Collagen I (Trevigen Inc., MD, USA), and were injected s.c. into the left flanks of the nude mice with 5 × 10^5^ or 5 × 10^4^ different cells. Six mice/group were used in the study. The engrafted mice were monitored twice a week for signs of tumor growth by measuring two perpendicular diameters of the tumors using calipers. The mice were sacrificed when their tumors were over 1.4 cm in the largest diameter [20].

### Preparation of 3D BME scaffold and cell growth standard curve

3D Culture BME Cell Proliferation Assay Kit (Trevigen Inc., MD, USA) was used in the assay. Briefly, BME gel was thawed on ice overnight in at 4°C; 35 μL of 3D BME scaffold was seeded into 96-well plates and was then transferred to a CO_2_ incubator set at 37°C for 60 min to promote gel formation. 2.5 × 10^5^ cells/mL cells were seeded into a pre-warmed medium containing 2% BME. Approximately 2.5 × 10^4^ cells were added on the top of the gel plug in each well. The cells were then mixed and incubated at 37°C for 96 hours. According to the manufacturer’s protocol, 15 μL of 3-D Culture Cell Proliferation Reagent (Trevigen Inc., MD, USA) was added to each well for continuous incubation at 37°C to produce a standard cell growth curve. The absorbance was read at 450 nm 1 to 4 hours after the addition of the reagent [21,22].

### Chemotherapeutic sensitivity in 3D *in vitro* environment

This assay tested the ability of 5FU (Sigma-Aldrich, Missouri, USA), DXT (Norzer Pharmaceutical Co. Ltd, Beijing, China), CDDP (Sigma-Aldrich, Missouri, USA) and Carboplatin (CBP, Sigma-Aldrich, Missouri, USA) to induce cell death after the establishment of the 3D cell culture. 2.5 × 10^3^ CD44^+^CD117^+^CSCs were resuspended in 150 μL 3D BME scaffolds that contained 17.18 μg/mL 5FU, 72 μg/mL DXT, 18.6 μg/mL CDDP and 20.5 μg/mL CBP, respectively. The drug concentrations taken in IC_50_ were based on our previous study of SKOV-3 cells and CD44^+^CD117^+^CSCs grown in 2D monolayer cells. To evaluate the effect of high concentration drugs on the CD44^+^ CD117^+^CSCs, two-folds and ten-folds of IC_50_ drug concentration were concurrently assayed. As a control, the chemotherapeutic sensitivities to various drugs were done in the 2D monolayer system. Chemotherapeutic sensitivity was detected by 3-(4,5- dimethylthiazol-2-yl)-2,5-diphenyltetrazolium bromide colorimetry assay after 24 hours of incubation.

### PTX induced cell apoptosis in 3D cell culture

When CD44^+^CD117^+^CSCs and SKOV-3 cells grew well in the 3D cell culture, 100 μL PTX (Econstar pharma. Beijing, China) at concentration of 100 μmol/L was added to each well for incubation at 37°C for 24 hours in a CO_2_ incubator. Caspase-3 and -7 activities in cell-based assays were detected using Caspase-Glo 3/7 Assay Kit (Promega Corporation, WI, USA). According to the manufacturer’s protocol, 100 μL of Caspase-Glo® 3/7 Reagent was added into a 96-well plate containing 100 μl of 3D BME scaffold, control cells or treated cells in the 3D cell culture. The content of the wells was gently mixed using a plate shaker at 300 rpm for 20 min, and was then incubated the plate at room temperature for 3 hours. The luminescence of each sample was measured by a Tecan GENios Pro reader (Tecan Australia Pty Ltd, Melbourne, Austria) as directed by the protocol.

### Cellular survival test in 3D cell culture

CD44^+^CD117^+^CSCs and SKOV-3 cells were cultured in the 3D BME scaffold for 2 weeks. During this period, the culture medium was changed every 6 days, and the cells were kept in the hungered situation for cellular survival test detected by using Cell Titer-Glo® Luminescent Cell Viability Assay Kit (Promega Corporation, WI, USA). The test was used to determine the number of viable cells in the culture based on the quantity of the ATP present, which signals the presence of metabolically active cells. 100 μL of CellTiter-Glo® Reagent equal to the volume of 3D cell culture medium was added to each well; the contents were mixed for 20 min to induce cell lysis, and the plate was incubated at room temperature for 30 min to stabilize luminescent signals, and then luminescence was recorded.

### RNA isolation and quantitative RT-PCR (qRT-PCR)

Total cellular RNA was extracted from 1 × 10^6^ SKOV-3 cells or CD44^+^CD117^+^CSCs by using Rneasy Mini Kit (Qiagen, CA, USA) by following the manufacturer’s instructions. qRT-PCR was performed using FastStart Universal SYBR Green Master with the LightCycler 2.0 real-time PCR system (Roche Germany). All reactions were performed in a 20 μL volume [23]. The PCR sense primer sequence for ABCG2 gene was 5^′^-GGCTTATACGGCCAGTTCCA-3^′^ and the anti-sense was 5^′^-GTCCGTTACATTG AATCCTGGAC-3^′^. The primer specific for the ABCB1 gene was 5^′^-CGAATGTCT GAGGACAAGCCAC-3^′^ and the anti-sense was 5^′^-CCATGAGGTCCTGGGCATG-3^′^. Primer specific for the Nanog gene was 5^′^-GGGCCTGAAGAAAACTATCCATCC-3^′^ and the anti-sense was 5^′^-TGCTATTCTTCGGCCAGTTGTTTT-3^′^[24]. The primer sequence for the human β-actin gene was 5^′^-GGACTTCGAGCAAGAGATGG-3^′^ and the anti-sense was 5^′^-AGCACTGTGTTGGCGTACAG-3^′^.

### Statistical analysis

Statistical analysis was performed using the Student’s *t*-test for the difference between the experimental groups and control group. Bonferroni correction was used where multiple comparisons were made. The differences were considered as statistically significant when P values were less than 0.05.

## Results

### Morphologic characteristics and phenotype identification of CD44^+^CD117^+^cells

**Figure 1:**
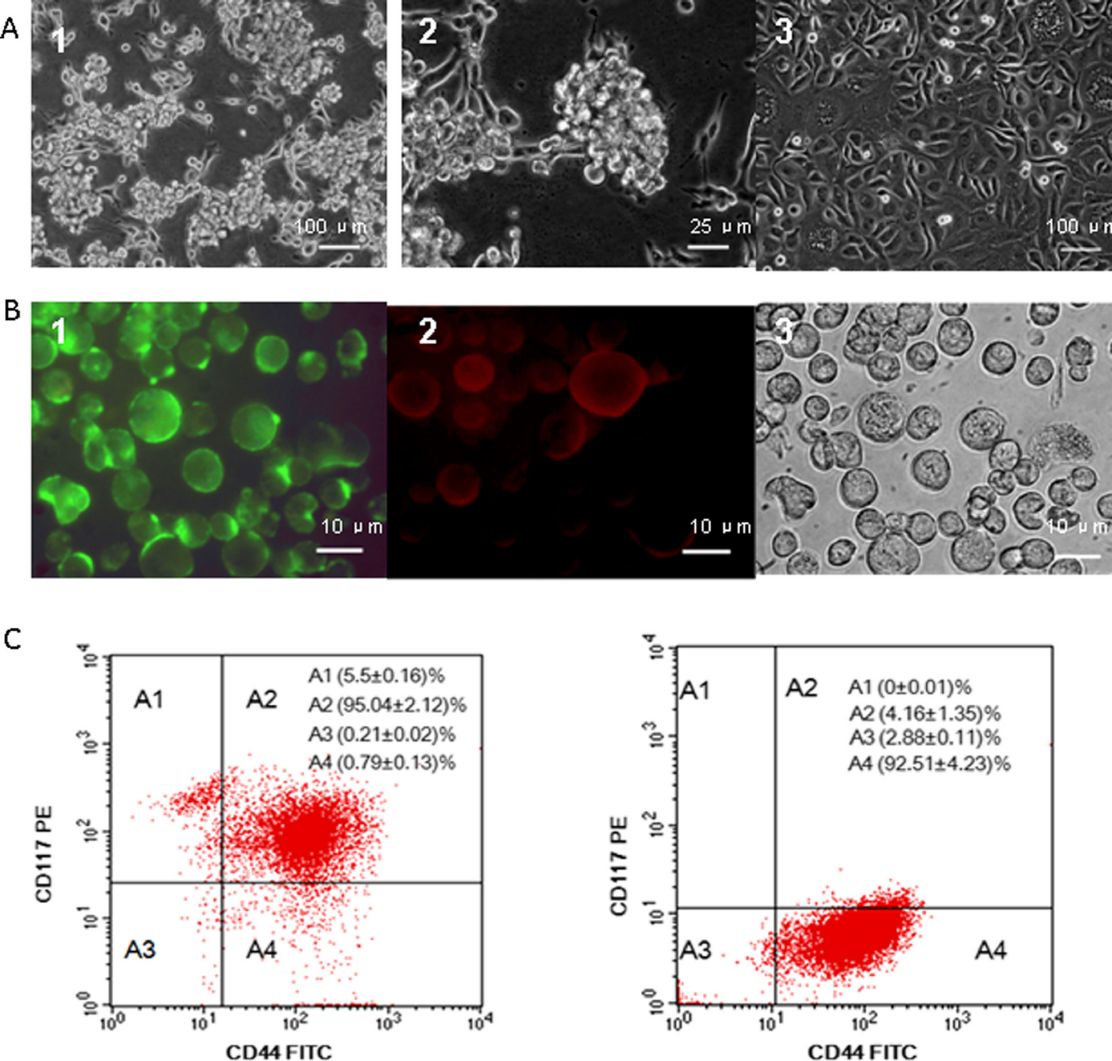
**Observation and analysis of CD44**^**+**^**CD117**^**+**^**CSCs. **The CD44^+^CD117^+^cells were sorted from the SKOV-3 cell line by MACS, and cultured in the stem cell culture medium on day 20 passage (A1, 100×, A2, 400×) or common 2D culture on day 10 passages (A3, 100×). B1 and B2 show the CD44 and CD117 positive cells detected by FCM. B3 displays the image of CD44^+^CD117^+^CSCs without staining with immunofluorescence (400×). Consistent with the result of immunofluorescence analysis, FCM analysis shows a high percentage of CD44 and CD117 molecules (95.04%) in the CD44^+^CD117^+^cells after being in the stem cell culture medium for 2 day (C Left); the percentage was only 4.16 for the SKOV-3 cells (C Right).

First, to avoid the differentiation of EOC CD44^+^CD117^+^cells in the common cell medium, the CD44^+^CD117^+^cells isolated from the SKOV-3 cell line by MACS were cultured in a stem cell culture medium. Figure 1A shows nonadherent spherical clusters of the CD44^+^CD117^+^ cells in ten days after plating under the stem cell culture condition. These cluster cells were larger, symmetric, and non-adherent (A 1, A2). Most spherical clusters were larger than 100 μm in diameter. In the in common cell medium (supplemented with 10% fetal bovine serum), however, the CD44^+^CD117^+^cells were differentiated, and did not form non-adherent spheres as shown in Figure 1A3. Second, the phenotype molecules of the EOC CD44^+^CD117^+^cells were identified by fluorescence microscopy. Figure 1B displays the CD44^+^ cells (B1), the CD117^+^ cells (B2) and the CD44^+^CD117^+^cells (B3) without immunofluorescence staining. Third, the CD44^+^ CD117^+^cells in the 2D stem cell culture were analyzed by FCM. Figure 1C exhibits 95.04% of the CD44^+^CD117^+^ cells (Left) on the 2nd day after isolation. In contrast, there were only 4.16% CD44^+^CD117^+^ molecules (Right) was found on the surface of the SKOV-3 cells. The results suggested that the MACS was a feasible, reliable method.

### Tumorigenic capability of CD44^+^CD117^+^cells in xenograft mice

**Figure 2:**
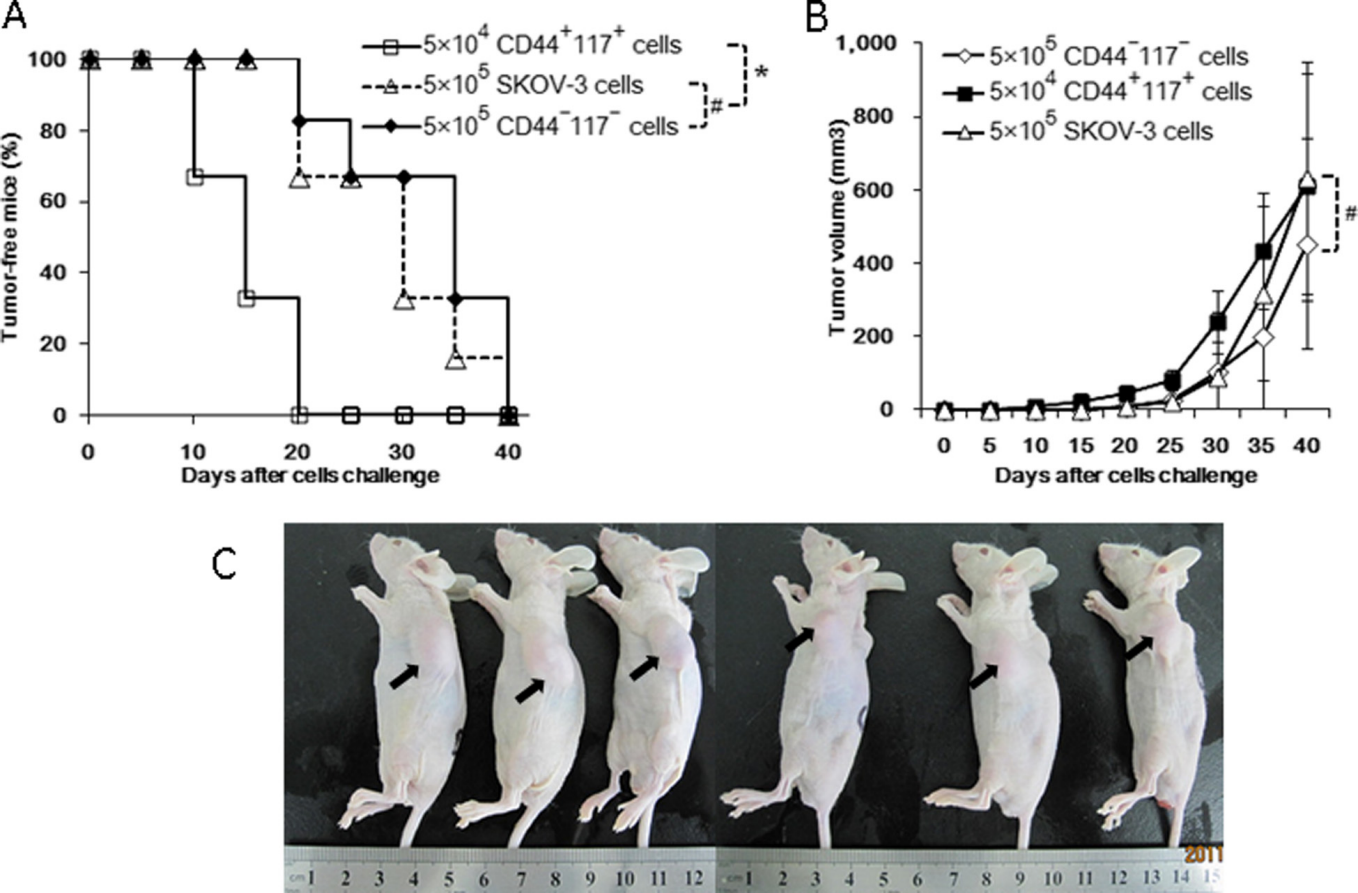
**Tumor growth in a xenograft mice injected with the different SKOV3 cells. A** shows the oncogenicity in mice s.c. challenged with 5 × 10^5^ CD44^−^CD117^−^ cells or SKOV-3 cells or 5 × 10^4^ CD44^+^CD117^+^cells, respectively. **B** indicates the tumor volume of the mice after being injected with the different SKOV-3 cells. **C** exhibits the images of tumor-bearing nude mice at 6 weeks after being injected with 5 × 10^4^ CD44^+^CD117^+^cells.

To demonstrate if the CD44^+^CD117^+^cells isolated from SKOV-3 cell line possessed powerful tumorigenicity, we designed the experiment, in which the CD44^+^CD117^+^cells were implanted into the athymic nude mice to observe the tumorigenicity of the cells. Figure 2A indicates that 2 of the 6 mice injected with 5 × 10^4^ CD44^+^CD117^+^cells could develope tumors on Day 10, and all the 6 mice developed tumors by Day 20. However, only 2 of the 6 mice injected with 5 × 10^5^ CD44^−^CD117^−^cells or the SKOV-3 cells began to form tumors on Day 22 and Day 20 in order, and all mice developed tumors in 40 days. No tumor development was, however, found in the 6 mice injected with 5 × 10^4^ CD44^−^CD117^−^cells or SKOV-3 cells until 50 days into the observation (data no shown). Figure 2B represents clinical pictures on Day 42 after the mice were injected with the 5 × 10^4^ CD44^+^CD117^+^cells. The tumor volume is shown in Figure 2C. There was no statistically significant difference (#*P > 0.05*) in tumor volume among the groups of the mice that were injected with different cells. Only the group that had 5 × 10^4^ CD44^+^CD117^+^cells injected into the mice, did develop tumors in the about 10 days. It is thus evident that the CD44^+^CD117^+^cells had strong tumorigenicity in xenografts *in vivo* in contrast to the CD44^−^CD117^−^cells or the SKOV-3 cells. These findings demonstrated that the CD44^+^CD117^+^cells possess the characteristics of EOC CSCs that have a strong tumorigenicity *in vivo*.

### Growth trait of CD44^+^CD117^+^CSCs in 3D culture model

**Figure 3:**
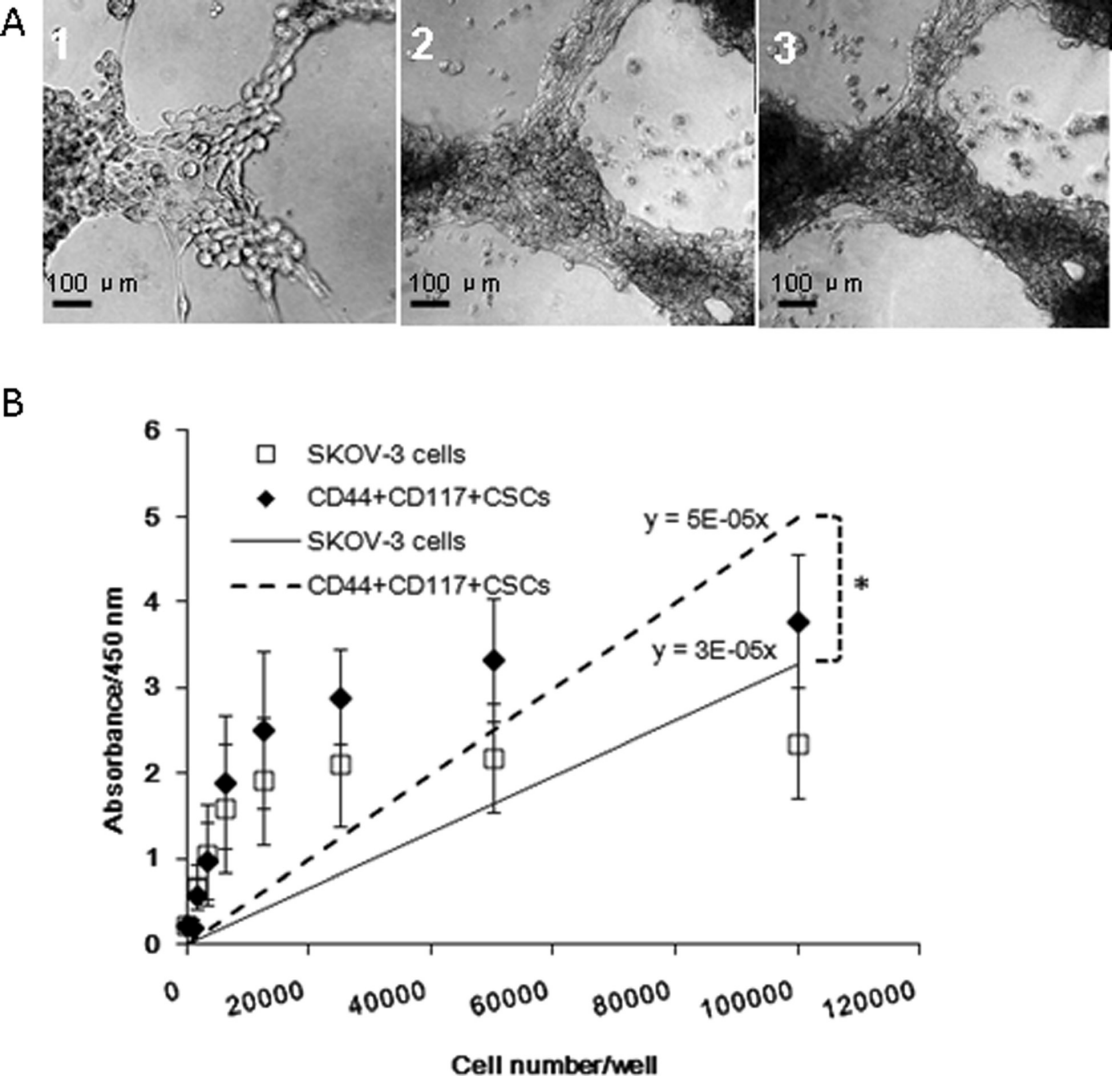
**Cell growth in 3D BME scaffold. **The CD44^+^CD117^+^CSCs grew more quickly in **A**1(2 days), A2(4 days) and A3(6 days). 100×. **B** shows standard growth curves of the CD44^+^117^+^CSCs and the SKOV-3 cells. Statistically significant differences are denoted by asterisk (*) for *P < 0.05*.

As is shown in Figure 1A, the CD44^+^CD117^+^ cells and the SKOV-3 cells in 2D culture model had slow growth activity; the cells form larger, nonadherent clusters in the 2D stem cell culture medium after 20 day culture. In the 3D culture model, however, the CD44^+^CD117^+^ CSCs grew faster than the SKOV-3 cells as is shown in Figure 3B. The cells were closed to one another on Day (Figure 3A1), the cell density was increased on Day 4 (Figure 3A2), and the cells formed distinctive clusters Day 6 (Figure 3A3). Eight days after being under the 3D culture condition, the cell cluster density increased continuously until there was not enough of the BME scaffold for cell growth (data no shown). At this stage, the cell density inside the scaffold may have reached the highest level. However, the oxygen and nutrient supply by passive diffusion may have no longer been able to meet the need of the cell growth, nor to support the cell clusters to grow any more. As a consequence, the CD44^+^CD117^+^CSCs cultured in the 3D BME scaffold for 6 days actively exhibited their proliferative potential, which suits researcher to investigate the tumor biological properties during the days.

### Analysis of CD44^+^CD117^+^CSC chemoresistance to anticancer drugs in 3D culture versus 2D one

**Figure 4:**
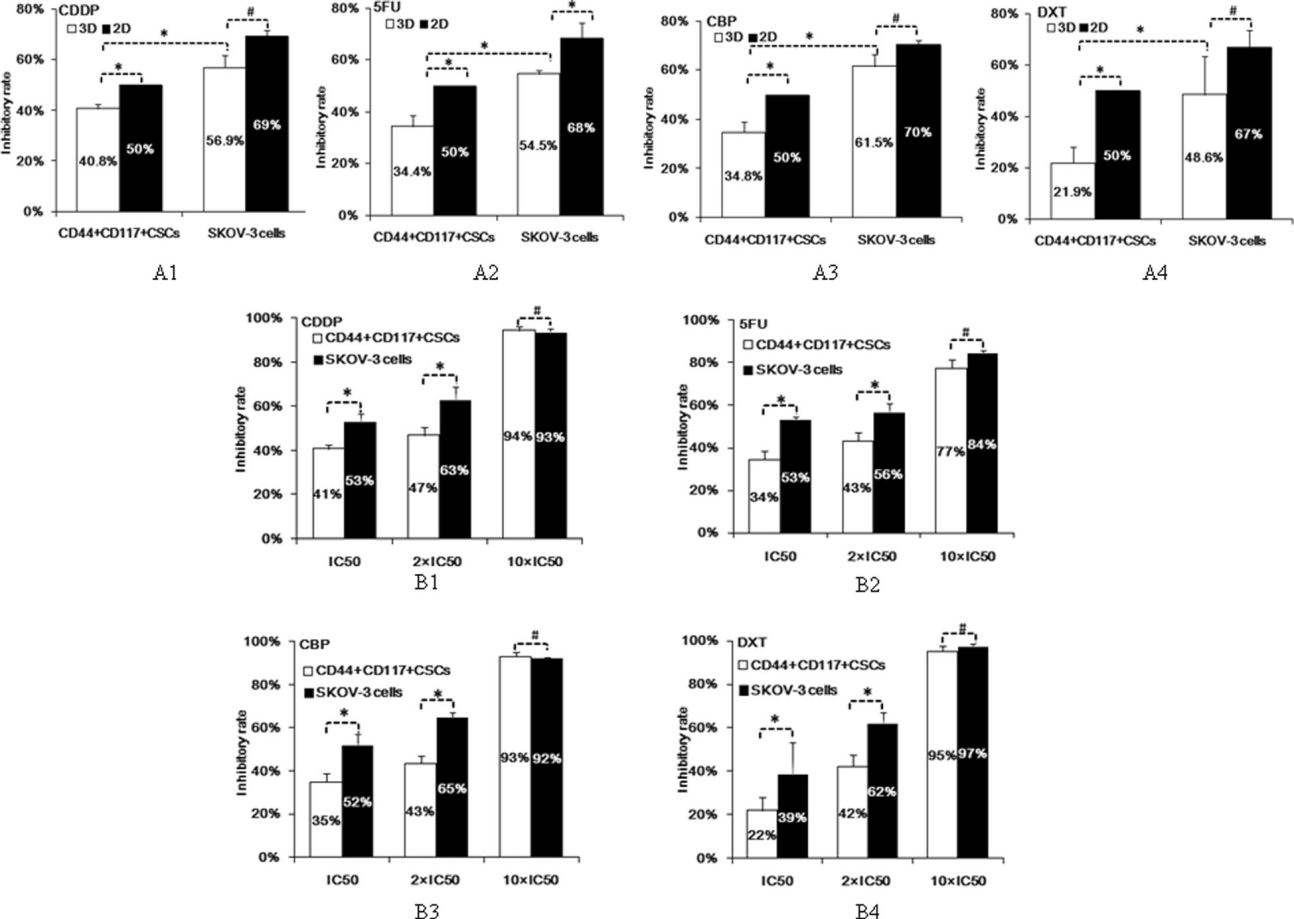
**CD44**^**+**^**CD117**^**+**^**CSCs with multidrug resistance property in 3D culture. A** shows the cellular growth inhibitory rates of anticancer drugs 5FU, CDDP, CBP, and DXT to the CD44^+^CD117^+^CSCs and the SKOV-3 cells in 3D culture and the 2D one, respectively. IC_50_ concentrations of 5FU, CDDP, CBP and DXT were 17.18 μg/mL, 18.6 μg/mL, 20.5 μg/mL, and 72 μg/mL, respectively, in the CD44^+^CD117^+^CSCs group. **B** likewise indicates the inhibitory rates of 5FU, CDDP, CBP, and DXT to CD44^+^CD117^+^CSCs and SKOV-3 cells in IC_50_ concentration, two-folds IC_50_ concentration and ten-folds IC_50_ concentration, respectively. * *P < 0.05* and ^#^*P > 0.05.*

To examine whether the CD44^+^117^+^CSCs possessed a hypothesized chemoresistance to drugs, we assessed the inhibitoryx rates of a panel of drugs to the CD44^+^117^+^CSCs and SKOV-3 cells; these drugs are commonly used in EOC chemotherapy and included 5FU, DXT, CDDP, and CBP. Between the 3D and 2D cultures with IC_50_ concentration, the CD44^+^CD117^+^CSCs showed a higher level of resistance to all the four drugs in the 3D culture than in the 2D culture. On the other hand, the SKOV-3 cells did not show statistically significant differences (#*P > 0.05*) between the 3D and the 2D cultures in resistance to the drugs. These results are shown in Figures 4A1-A4.

Additionally, since high-dose chemotherapy is regularly employed in clinical EOC therapy, we wanted to know if such effects can be reproduced in the 3D cultured CD44 + CD117+ CSCs with high doses. Accordingly, we designed an experiment in which the CD44^+^117^+^CSCs were incubated in the 3D culture with each of the four drugs with two-folds and ten-folds of IC_50_ concentration, respectively; the sensitivity of the cells to each drug was evaluated. It was found that the CD44^+^ CD117^+^CSCs were still more resistant in the 3D culture than in the 2D culture to all the four drugs with the two-folds of IC_50_ concentration. With the ten-folds of IC_50_ concentration, however, hardly any CD44^+^117^+^CSCs or SKOV-3 cells survived in the 3D culture; there was no statistically significant difference between the CD44^+^117^+^ CSCs and the SKOV-3 cells in Figures 4B1-B4.

### Apoptosis and survival of CD44^+^117^+^CSCs in 3D culture versus 2D one

**Figure 5:**
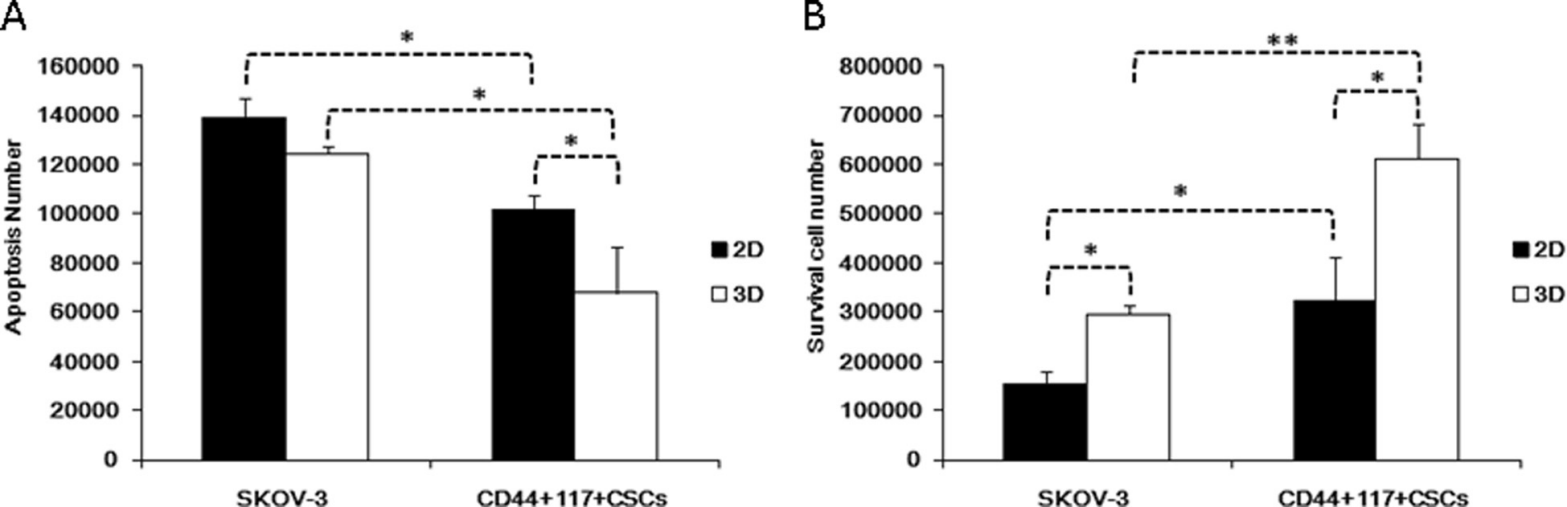
**Less apoptosis and more survival of CD44**^**+**^**117**^**+**^**CSCs than SKOV-3 cells in 3D culture versus 2D one. A** shows the cell apoptotic results detected by Cell Titer-Glo® Luminescent cell viability measurement. **B** indicates the survival cell numbers in the 3D and 2D culture respectively, from the test by Survival assay. Statistically significant differences are indicated by asterisk (*) for *P < 0.05* or (**) for *P < 0.03*.

PTX is one of the best antineoplastic drugs found from in the nature in the past decades and has been shown to be effective against EOC through induction of cell apoptosis [25]. To test the effect of PTX on the CD44^+^117^+^CSCs in the 3D cell culture, we employed Caspase-Glo® 3/7 Assay in the study. Figure 5A suggests that fewer CD44^+^117^+^CSCs than the SKOV-3 cells underwent apoptosis in the 3D culture medium containing 100 μmol/L PTX. In contrast, the CD44^+^117^+^CSCs that were cultured in the 2D environment containing the same concentration of PTX did not demonstrate the same amount of efficacy as the CD44^+^117^+^CSCs cultured in the 3D culture. It was hypothesized above for Figure 3 that the tumor tissues in the 3D culture were in an innutritious and oxygen deficient microenvironment due to cancer cell fast growth and the cells were unable to survive in this microenvironment. To test this hypothesis, we performed the cell survival assay and assessed the viability of the CD44^+^117^+^CSCs in the hypoxia stress microenvironment. When the cells were cultured in the 3D environment for 6 days, more apoptosis of SKOV-3 cells observed than that in the 2D environment. Nevertheless, more CD44^+^117^+^CSCs exhibited pertinacious survival potential in 3D culture than in the 2D culture (Figure 5B).

### Analysis of mechanism of CD44^+^117^+^CSC drug resistance

**Figure 6:**
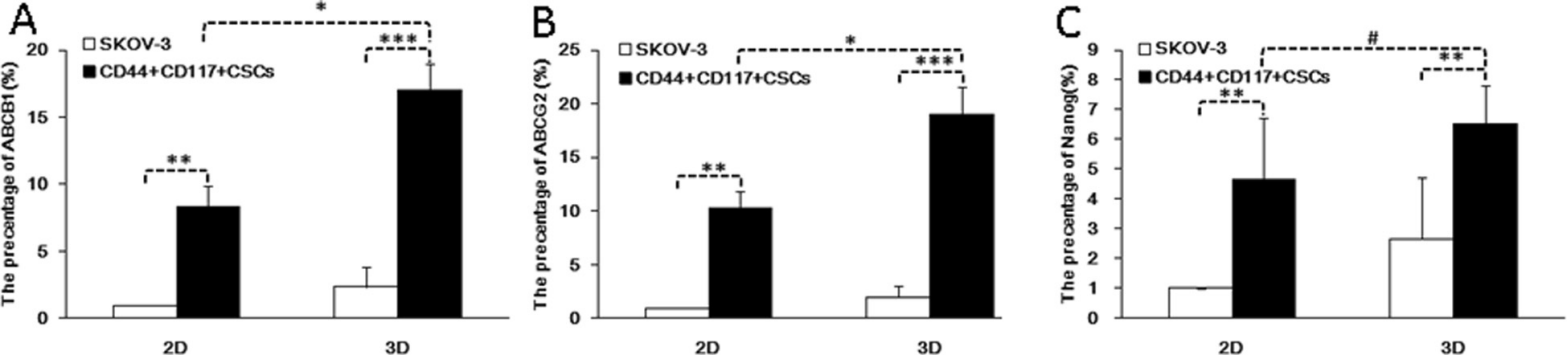
**Expression of ABCG2, ABCB1 and Nanog in the CD44**^**+**^**117**^**+**^**CSCs in 3D culture and 2D cultures. **A-C indicate the expression of ABCG2, ABCB1 and Nanog in the CD44^+^117^+^CSCs and SKOV-3 cells respectively, as they were cultured in the 3D or 2D culture medium detected by qRT-PCR. Statistically significant differences are indicated by asterisk (*) for *P <0.05,* (**) for *P < 0.03,* (***) for *P < 0.01.* # indicates *P > 0.05*.

Drug-resisting tumor cells are known to over express one of several ATP-binding cassette (ABC) transporters that include multidrug resistance1 (MDR1), ABCB5, and ABCG2 etc [26]. Our previous studies have demonstrated that the ovarian cancer cell lines of SKOV-3 and A2780 expressed transporters of ABCG2 and ABCB1 that were closely associated with the drug resistance [23,27]. To further evaluate the effect of ABCG2 and ABCB1 on drug resistance by the CD44^+^117^+^CSCs, we detected the molecular expression on the CD44^+^117^+^CSCs cultured in the 3D and 2D media, respectively. The results of qRT-PCR showed that the expression of ABCG2 and ABCB1 on the CD44^+^117^+^CSCs were higher than that on the SKOV-3 cells in the 3D and 2D media (Figures 6A- 6B); the differences were statistically significant (*p* < 0.05). However, no visible difference was found in the expression of the Nanog molecule (#*P > 0.05*), which was associated with the differentiation of stem cells, between the 3D and the 2D cultures, respectively, even though the expression of Nanog was significantly upregulated in the CD44^+^117^+^CSCs compared with the SKOV-3 cells in 3D and 2D cultures (*p* < 0.01); the results are shown in Figure 6C.

## Discussion

The existence of EOC CSCs might explain why standard chemotherapy may shrink tumors; however, most tumors of this highly lethal gynecologic malignancy re-grow and eventually cause a relapse [28,29]. Therefore, it is necessary to investigate the molecular mechanisms of EOC CSC chemoresistance. However, cancer investigation in the premise of “cancer as a cell based-disease” focused on finding tumor suppressor genes and oncogenes, while the role of the cancer cell environment was neglected [30]. In our current study, we focused on the microenvironment of EOC CD44^+^CD117^+^CSCs and evaluated the characteristic of CD44^+^CD117^+^CSCs in the 3D model versus the 2D model.

We firstly demonstrated that the CD44^+^CD117^+^cells that were isolated from the SKOV-3 ovarian cell line and were identified by immunofluorescence microscope and the FCM (Figure 1) possessed the CSC properties. This is because the CD44^+^CD117^+^CSCs had powerful tumorigenic capability in the xenograft mice (Figure 2) and had fast growth activity in the 3D model versus in the 2D one when compared with the SKOV-3 cells (Figure 3). These data provided authentic evidence that the CD44^+^CD117^+^cells were EOC CSCs, and established a foundation for further evaluation of EOC CSC chemoresistance in the 3D model versus the 2D one.

It is known that there have existed significant discrepancies between the efficacy of potential anticancer drugs when they are tested *in vitro* using cancer cells generally grown in monolayer cells and the clinical application of these drugs they are evaluated *in vivo*[31]. For this reason, we employed the 3D culture based on the BME scaffold that is more adequate for analyzing the efficacy of anticancer drugs was employed in the present study. Because 5FU, CDDP, CBP, DXT and PTX were standard chemotherapeutic drugs for EOC, we selected these drugs to investigate their effect on the CD44^+^CD117^+^CSCs. The adopted IC_50_ concentration was based on our previous experiments (data no shown here). The results in Figure 4 demonstrate the discrepancy in the effects of 5FU, CDDP, CBP and DXT between the 3D model and the 2D one. 5FU, CDDP, CBP and DXT inhibited CD44^+^CD117 ^+^CSC growth by 50% in the 2D culture with the IC_50_ concentration; the effects of the drugs on the CD44^+^CD117^+^CSCs in the 3D model was significant lower than the effects of the drugs on the monolayer cells in the 2D model; 5FU, CDDP, CBP and DXT inhibited the growth of CD44^+^CD117 ^+^CSCs by only 34.4%, 40.8%, 34.8% and 21.9%, respectively. The standard 2D cell culture condition drastically differs from that in the 3D one. Our findings may help to explain why the effect of some anticancer drugs have demonstrated valid effects on cancer cells when evaluated *in vitro* using 2D cell culture system, but the drugs have shown significant discrepancies in the observed efficacy when these drugs are evaluated *in vivo*[15].

In the study, we also found that the CD44^+^117^+^CSCs grew very slowly in the 2D culture and needed more than 20 days to form large, nonadherent clusters. Surprisingly, the CD44^+^117^+^CSCs grew fast in the 3D BME scaffold and formed a distinctive cell clusters on day 2, and developed the tumor spheres on day 4. However, this was not observed in the SKOV-3 cells. The property of fast growth observed in the CD44^+^117^+^cells in 3D BME scaffold was consistent with the characteristic of CSCs *in vivo*, which was reflected in the tumorigenic capability of the xenograft mice (Figure 2).

PTX is known to have induced the G1 arrest and early apoptosis of cancer cells in the treatment of ovarian cancer [32]. Consequently, more SKOV-3 cells underwent apoptosis than CD44^+^117^+^CSCs in the 3D and 2D culture systems, respectively. However, when the CD44^+^117^+^CSCs and SKOV-3 cells were cultured in the 2D environment, the effect of PTX on these cell survival was not as obvious as in the 3D culture (Figure 5B). The results suggested that the compared to the 2D culture, the 3D culture reflected the tumor cells *in vivo* response to PTX therapy more objectively.

Evidence from recent research has shown that the oxygen and nutrient supply in the center of the tumor tissue are not enough to satisfy the need of cells of the tumor tissues, and this hypoxic microenvironment would make it hard for the tumor cells to survive [33,34]. Thus, the data of survival assay showed that the SKOV-3 cells in 3D BME scaffold were hardly to keep long time and gradual apoptosis compared with the CD44^+^117^+^CSCs that were induced to adapt the hypoxic and innutritious environment in the 3D structure. This finding was consistent with CSCs in tumor tissues *in vivo*[34].

In this study, we observed differences in the efficacy of the anticancer drugs, the growth activity of the cancer cells, and the survival potency in the 3D and the 2D models; these differences led us to investigating the mechanism of the CD44^+^117^+^CSCs’ resistance to chemotherapeutic agents when the CD44^+^117^+^CSCs were cultured in the 3D and the 2D environments. Figure 6 indicates that the expression of ABCG2 and ABCB1 on the CD44^+^117^+^CSCs were more significantly upregulated in 3D environment compared to 2D one in presence of the 5FU, CDDP, CBP and DXT, respectively. According to the CSC hypothesis [35], CSCs are naturally resistant to chemotherapy through the expression of ABC-transporter that enables a cancer to escape the cytotoxic effects of chemotherapy, which might be one of important mechanism in the development of resistance to chemotherapy. Compared with the SKOV-3 cells, the CD44^+^117^+^CSCs not only markedly increased the expression of ABCG2 and ABCB1 in the 2D environment compared with the SKOV-3 cells, but also enhanced their expression in the 3D BME scaffold in contrast to the 2D environment. We analyzed the BME scaffold that formed the elaborate 3D microenvironment that was similar to the tumor *in vivo* and facilitated the maintenance of the inherent malignant trait of CD44^+^117^+^CSCs, and this is maybe one of the reasons that CD44^+^CD117^+^CSCs were more resistant to 5FU, CDDP, CBP, DXT and PTX in the 3D environment than in the 2D environment at IC_50_ concentration. However, the expression of Nanog molecule in the CD44^+^CD117^+^CSCs was not the same as the expression of ABCG2 and ABCB1 in the 3D culture system. Because the Nanog molecule is transcriptional determinants and directs the multi-potential differentiation of undifferentiated stem cells [24,36], it may be not closely associated with the resistance to chemotherapy.

## Conclusion

Our findings from this study demonstrated definitive evidence of the existence of CD44^+^CD117^+^ CSCs in the human SKOV-3 ovarian cell line. In the 3D culture system, EOC CD44^+^ CD117^+^CSCs showed stronger chemoresistant to 5FU, CDDP, CBP, and DXT, higher proliferative potential and faster growth activity as well as more resistance to apoptosis induced by PTX than in the in 2D cell culture system. To our knowledge, this is the first study of characteristics of EOC CD117^+^CD44^+^CSCs in 3D culture versus 2D one. This work will provide promising insights into the development of novel anticancer drugs targeted to EOC CSCs in the 3D BME scaffold culture model *in vitro*.

## Abbreviations

3D: Three dimensional; 2D: Two-dimensional; CSCs: Cancer stem cells; EOC: Epithelial ovarian cancer; ABC: ATP- binding cassette; BME: Basement membrane extract; CBP: Carboplatin; CDDP: Cisplatin; DMEM: Dulbecco’s Modified Eagle Medium; DXT: Docetaxel; FCM: Flow cytometer; MACS: Magnetic associated cell sorting; MDR1: Multidrug resistance1; PTX: Paclitaxel; qRT-PCR: Quantitative RT-PCR.

## Competing interests

The authors declare that they have no competing interests.

## Authors’ contributions

Junsong Chen, Jing Wang and Dengyu Chen carried out the experiments described in the manuscripts, developed the technique described in the manuscript, and participated in the writing of the manuscript. Jie Yang, Cuiping Yang, Yunxia Zhang and Hongyi Zhang participated in most of the experiments. Jun Dou contributed to the design of the experiments and to the writing of the manuscript. All authors have read and approved the final manuscript.

## Authors’ information

Co-authors: Junsong Chen, Jing Wang and Dengyu Chen
